# The perception of the hidden curriculum on medical education: an exploratory study

**DOI:** 10.1186/1447-056X-8-9

**Published:** 2009-12-15

**Authors:** Manabu Murakami, Hidenobu Kawabata, Masaji Maezawa

**Affiliations:** 1 Department of Healthcare Systems Research, Graduate School of Medicine, Hokkaido University, Sapporo, Japan

## Abstract

**Background:**

Major curriculum reform of undergraduate medical education occurred during the past decades in the United Kingdom (UK); however, the effects of the hidden curriculum, which influence the choice of primary care as a career, have not been sufficiently recognized. While Japan, where traditionally few institutions systematically foster primary care physicians and very few have truly embraced family medicine as their guiding discipline, has also experienced meaningful curriculum reform, the effect of the hidden curriculum is not well known. The aim of this study is to identify themes pertaining to the students' perceptions of the hidden curriculum affecting undergraduate medical education in bedside learning in Japan.

**Methods:**

Semi-structured interviews with thematic content analysis were implemented. Undergraduate year-5 students from a Japanese medical school at a Japanese teaching hospital were recruited. Interview were planned to last between 30 to 60 minutes each, over an 8-month period in 2007. The interviewees' perceptions concerning the quality of teaching in their bedside learning and related experiences were collected and analysed thematically.

**Results:**

Twenty five medical students (18 males and 7 females, mean age 25 years old) consented to participate in the interviews, and seven main themes emerged: "the perception of education as having a low priority," "the prevalence of positive/negative role models," "the persistence of hierarchy and exclusivity," "the existence of gender issues," "an overburdened medical knowledge," "human relationships with colleagues and medical team members," and "first experience from the practical wards and their patients."

**Conclusions:**

Both similarities and differences were found when comparing the results to those of previous studies in the UK. Some effects of the hidden curriculum in medical education likely exist in common between the UK and Japan, despite the differences in their demographic backgrounds, cultures and philosophies.

## Background

In the United Kingdom (UK), significant curriculum reforms occurred, in which the UK General Medical Council emphasized reducing information overload of medical knowledge and diverging from didactic teaching [1]. Nevertheless, little attention was paid to the hidden curriculum [1], the effect of which influences students and faculty most concerned about primary care careers [2]. In his interview published in *Parade Magazine *in 1994, C. Everett Koop, the former U.S. Surgeon General, cited "...What leads most young doctors away from such practice [primary care] is not money but a medical school culture that devalues the family doctor." [2,3]

Most of what is acquired in medical school education contributes not to the manifested offerings called formal curriculum (defined by Hafferty as the stated, instead, and formally offered and endorsed curriculum [4,5]) but to the learning process called hidden curriculum (defined as a set of influences that function at the level of organizational structure and culture [4,5]). The discordance between what formal curriculum intends to teach the medical students and what students perceive to be learned from hidden curriculum is difficult to deal with. It seems to be illustrated explicitly in the example of the white coat ceremony as a curricular event, which was originally expected to symbolize the students' sense of compassion and humility, but instead serves to impress care-giving hierarchies and the social privileges of physicians [6], though this might be one of its functions. If advocates for educational reforms have never been aware of solutions to the problem of reconciling the discordance between these curricula, "reform without change" may occur, as history repeats itself [7]. That is perhaps why it has only been over the past decades that researchers of medical education have come to focus on the concept of hidden curriculum [8]. Lempp & Searle presented a qualitative study of medical students' perceptions of teaching in light of undergraduate medical education in the UK, which conveyed critical problems about latent features of the effect of the hidden curriculum on the realm of medical education [1].

In the meantime, aspects of educational curriculum in Japanese medical schools have also undergone a major transformation over the past decades. Information about this, including problem-based learning and model core curriculum in Japanese medical education, was available elsewhere [9-11]. Such unprecedented curriculum change has never been experienced in Japan in the Postwar period.

The prior study implemented by Lempp & Searle in the UK found these four themes about the perception of the hidden curriculum; "Personal encouragement," "haphazard teaching," "important of hierarchy," and "getting ahead by being competitive." However, no similar qualitative study concerning the perception of the hidden curriculum in Japanese medical schools has ever been conducted. Since many traditional Japanese medical schools adopted elements of the German style [12], which is quite distinct from the system in place in the UK, it is expected that many disparate themes may evolve from a similar survey in Japan.

Accordingly, to explore themes pertaining to the students' perceptions of the hidden curriculum of undergraduate medical education in bedside learning in a Japanese medical school, and compare them with the prior study results from the UK, was the purpose of this study. It would be useful to utilize the results of our survey as feedback on teaching methods in faculty development workshops, which will contribute to the reform of medical education in Japan.

Relating to the training for careers in family medicine in Japan, traditionally few institutions systematically foster primary care physicians and very few have truly embraced family medicine as their guiding discipline [13]. (Note: since Japan has few physicians trained in the delivery of primary care, and lacks recognition of the discipline of family medicine, here we use the term "primary care" to refer to the function as stated by the U.S. Institute of Medicine; that is, primary care specialties include family medicine by this definition [13].) Despite the shortage of primary care specialists, one of the authors [MMa] continued to emphasize at length how important primary care physicians' roles as medical educators was, and the necessity of community-oriented medical education in Japan [14].

## Methods

We conducted qualitative methodology employing 1-to-1 semi-structured interviewsand thematic content analysis [15-17] to elicit and perceive in-depth thoughts and learning experience. We used purposive sampling to identify a target population of undergraduate year-5 medical students in the initial stage of clinical training in a Japanese medical school. They were recruited as purposeful sampling to concentrate on the clinical stage (i.e. bedside training in clinical medicine).

**Figure 1:**
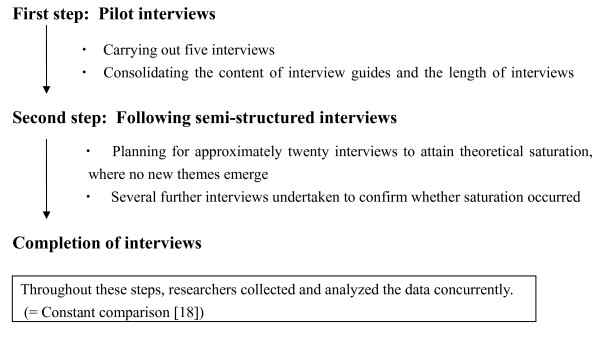
**Interview Process**. We planned to use two steps: pilot interviews (the first step) and following semi-structured interviews (the second step).

The interview agenda outlined in Figure 1 was conducted over an 8-month period in 2007, by the two researchers (MMu and HK) independently, with each interview lasting 30 to 60 minutes in a private room, taking confidentiality into account. At the start of each interview, we used broad and open-ended questions and gradually asked personal and in-depth questions, which had no threatening effect on students and gave the advantage of developing rapport with them.

A preliminary interview guide was created, referring to the prior study [1] and initial 5 pilot interviews. The final interview guide was as follows, (1) Introduction/Icebreaker: training atmosphere: *1. What do you think about your training, in general? 2. What do you enjoy most/least in your training? 3. Has the medical training met with your satisfaction? Why/Why not? *(2) Knowledge and skills of medical practice: *4. What technical/diagnostic skills and knowledge do you wish to acquire the most? 5. Do you reasonably expect to be able to attain your medical goal at this stage? Please explain why or why not*. (3) Career choice in medicine: *6. What career decision-making plans do you have? 7. What factors affect your choice of specialty? *(4) Impact of training on individual medical students' professional relationships: *8. What motivated/demotivated you to practice in your bedside learning? Could you provide some actual examples? 9. What difficulties did you experience in your training? Could you describe what happened? 10. What did you like/dislike about your contact with your mentors, paramedical staff, and colleagues? Could you give examples? *(5) At the end of the interview: *11. Finally, do you have any issues you want to add concerning clinical training? Is there anything else you think is important for your clinical training?*

All interviews were tape-recorded and transcribed, and all verbatim scripts were preserved for anyone to be able to replicate the findings in a similar environment and have a clear understanding of how the authors arrived at their conclusions. Interviews were carried out until no new themes emerged. Each medical student signed a written form of informed consent.

The scripts were read in their entirety and analysed separately as follows: Firstly, scripts were split into meaningful words, phrases or sentences and shortened while preserving the core quality of the scripts. We refer to these parts as "units". Secondly, these units were aggregated with similar groups of content and labeled "codes". Thirdly, using these codes, we created "categories" which were groups of content that share a commonality and are mutually exclusive and exhaustive. Finally "themes", which linked the latent meanings together in these categories, emerged.

The research interviews and analysis were conducted concurrently; that was called constant comparison [18], and preliminary results pertaining to these themes were presented to the third author (MMa), unfamiliar with the research processes, to validate and revise the findings. All three authors contributed to discussion, revised the results, and came to a final agreement.

All study process was implemented utilizing Japanese Microsoft Excel 2003 and later the results were translated into English. To ensure that the results are both cogent and reasonable, all interviews were painstakingly translated back into Japanese. Back translation both improves the reliability and validity of the research by requiring that the quality of the translation is verified by an independent translator, translating back into the original language [19].

## Results

Twenty-five (18 males and seven females, mean age 25 years old, demographic data was shown in Table 1) students consented to participate. All of them were interviewed willingly and seven main themes emerged: "the perception of education as having a low priority," "the prevalence of positive/negative role models," "the persistence of hierarchy and exclusivity," "the existence of gender issues," "an overburdened medical knowledge," "human relationships with colleagues and medical team members," and "first experience from the practical wards and their patients."

**Table 1:** Characteristics of study participants*

	year 5 medical students (n = 25)
Mean age	25
Sex	Male 18
	Female 7
Ethnicity	All Japanese (except one male student†)
Entry to medical school‡	After high school (including gap years) 25
	Obtained degrees besides M.D. 0
Marital status	Single 24
	Married 1
	Has children 1

*No characteristics were outside population norms.

†There was no difference in ethnicity: Except for one overseas male medical student from Nepal, all subjects were Japanese. (This had no significant effect on the results).

‡Like other tertiary education, medical school in Japan begins immediately after graduation from high school, but lasts 6 years [9].

We did not obtain religious information from the study participants in the interest of protecting their privacy. However, it is reasonable to assume that this had no significant effect on the data due to most Japanese being atheist.

### 1) The perception of education as having a low priority

In total, 14 out of 25 students stated that teachers tended to not infrequently ignore scheduled times for classes or canceled lectures without prior notice, which served to demotivate medical students. While some students accepted and tolerated a certain amount of laziness due to teachers' being busy with other work, they fervently appealed for giving notice in advance and not wasting students' time.

"*When they come, they seem to be running late for some other engagement, and make do with some brief handouts and a 10-minute lecture, after which they promptly leave, saying, 'you are dismissed.' "*

### 2) The prevalence of positive/negative role models

Most (20/25) medical students mentioned the positive or negative effect of mentors, which clearly illustrates a powerful influence. Some students (17/25) asserted that teachers' enthusiasm inspired them and even affected their career choice and development as doctors. Conversely, others (8/25) reported that negative role models had effectively demotivated the learners, which seemed to affect students more strongly than positive role models. As an example of what behavior demotivated students, one of the interviewees offered the testimony that a classmate attended only the final lecture, yet was penalized in no way whatsoever.

"Some doctors taught us both conscientiously and enthusiastically. They gave us detailed instructions that were most useful to us as learning aids. They served as a good model, and both impressed and motivated us."

### 3) The persistence of hierarchy and exclusivity

15 of the 25 medical students mentioned that they were treated unfairly for no other reason than their lower position in the ward. There were also numerous mentions of senior staffs' verbal abuse and vilification of other departments or institutions, causing a serious deterioration in the relations of interdisciplinary or multidisciplinary teams.

"They keep telling everyone awful stories about other departments and colleges. They have convinced themselves that theirs is the best way of doing anything. I'm totally sick of hearing about it."

### 4) The existence of gender issues

About half of the medical students (6/18 males, 6/7 females) described problems relating to the peculiarity of the conventional male-dominant position in Japanese medical society. Some female students complained of sensitive issues that were specific to women, which had to be rather difficult for men to understand. In addition, another participant gave witness to the fact that one female colleague had been subjected to sexual harassment.

"Men are in the majority. Well, I have severe menstrual pain ... But I'm too embarrassed to say that's why I can't continue bedside training on a given day. I'm sure that they can never understand how hard it is for me. Creating a supportive atmosphere for women is very difficult. And I don't think there's really much hope..."

### 5) An overburdened medical knowledge

Most (22/25) medical students mentioned that they must place writing their medical reports above their interactions with their patients due to their lack of learning time. Even if they hope to adopt holistic medicine, in which they embrace not only biomedical but also psychosocial approaches and focus on patient-centered care, they could not do that, since it was not as highly rated as the test marks.

"The only thing you must do to submit your reports is to make a copy of your textbooks."

### 6) Human relationships with colleagues and medical team members

All together, 10 of the 25 medical students in the same year of training considered themselves to be more cooperative, unlike their UK counterparts who reported highly competitive characteristics. Some medical students also mentioned that relaxation of the atmosphere at the department is very important to promote them to study harder. They seem to put emphasis on harmony with other members in their group.

"There are variations in how well skills are learnt, just as there are with individual drive and motivation, but as a group, we must do everything cooperatively."

### 7) First experience from the practical wards and their patients

About one third of (9/25) medical students reported that their patients' support and encouragement enhanced their motivation for studying. As they were getting more and more empathetically connected with their patients, they felt more confident perceiving the patients as not only a disease but a living person with a disease. Additionally, they seemed to feel glad when patients encouraged them to say that students were on their way to becoming a doctor and they would depend on them in the future.

"I gained deep insight into the aspects of my patients from not only a biomedical but also a psychosocial point of view. No doctor-patient relationship but an equal partnership exists here..."

## Discussion

We found both similarities and differences when comparing our results to those of previous studies in the UK. In Japan, qualitative studies investigating this theme published in English are few and far between, probably making this the first piece of research qualitatively exploring educational practices in Japanese medical schools.

The results indicate that at least some effects of the hidden curriculum in medical education likely exist in common in many so-called advanced countries. This is all the more intriguing when one considers that Japan adopted Confucian pedagogical principles from China, and a 19th century Western medical format from Germany [9], yet still exhibits the same effects from a hidden curriculum in medical education as the UK.

Regarding the themes, "the perception of education as having a low priority" and "an overburdened medical knowledge," as Wear pointed out in her article, one usually dominates the others when an institution has conflicting goals [20]. As is usual in such a case, the values of education are subordinate to those of research or the clinic, and due to outcome will have not been identified promptly. In such an outcome-burdened environment, great importance is attached not to process but to outcome [21], so it is understandable that many people including students and faculty members believe the aphorism: "knowledge is power," whereby medical education with information overload will be accepted.

In addition, as the themes, "the persistence of hierarchy and exclusivity," "the prevalence of positive/negative role models," and "the existence of gender issues," Japanese cultural and ethnic background may make these problems more complicated. As Lempp & Seale indicated, "shy and quiet female Asian students were more likely to be humiliated or ignored by consultants during ward rounds [22]." Besides, seniority carries a lot of weight in most Japanese medical schools, in which the *sempai *(senior)-*kohai *(junior) relationships are strongly emphasized. These traditional Japanese educational practices in medical schools may have had been responsible for problems related to hierarchical structure. Pertaining to the issue of persistence of hierarchy and exclusivity, for example, the teachers' behaviors, such as verbal abuse and vilification of other departments, seemed unlikely to give these instructors an advantage over others, and resulted only in serving to generate harsh criticism of the professors from the students. Gender discrimination and career obstacles for women still exist in Japan as well, as Kaneto suggested [23]. That's also a common problem for both western industrialized countries [24] and Japan.

Conversely, we discovered intriguing differences between the UK and Japan in two particular themes: "human relationships with colleagues and medical team members," and "first experience from the practical wards and their patients." Japanese medical students tend to have less time in hospitals on a practical basis due to the structure of the medical curriculum in Japanese medical universities [9-11]. According to the study of Lempp & Seale, competitive rather than cooperative characteristics were more common among clinical medical students [1], which does not necessarily apply to our Japanese cases. The Japanese approach towards education, which stresses didactic lectures and rote memorization -- that is to say, "teacher centered, examination-driven and passive learning," symbolizing an educational philosophy that is much like that of many East Asian nations, which TEO & Song et al found [9,25], may also have an effect on Japanese medical students. In other words, the seemingly favorable results of Japanese medical students noted in our study might be a by-product of curricular differences.

We have to take the points of contention below into account in this study. Firstly, further research from other medical schools to assure generalizability will be needed, as our research was conducted in only one medical school. Secondly, researchers' vantage points also have to be considered. Two of the authors (HK and MMa) are bedside training instructors and supervise all students, which might have influenced interviewees' responses -- they may not have given sufficiently candid opinions regarding this research. Finally, potential linguistic problems remain, namely, we can never convey certain subtle differences in translations from Japanese into English, even though the attempt for back translation was made. As suggestions for further studies, we suggest a longitudinal study for measuring medical students changing their points of view, and addition of other medical schools to the study. Based on the research results in this study, we must conduct more comprehensive, data-generating interviews and explore the students' thoughts, experiences and perceptions more deeply.

## Conclusions

In our study, 7 main themes emerged, and at least some effects of the hidden curriculum in medical education likely exist in common between the UK and Japan, despite the differences in their demographic backgrounds, cultures and philosophies. The authors are clarifying the whole picture of the effect of the hidden curriculum that is not easily perceived but deeply affects the students' behavior [21] in medical education. We conclude our paper with Hafferty's compelling suggestion, "There is much work left to be done, including the critical step of framing change in terms of restructuring learning environments rather than in terms of modifying curricula [5]". In the case of the learning and training environment for Japanese primary care physicians, as we stated, systematically few doctors have been fostered as role models due to the tendency towards specialist, as opposed to generalist, training. This negatively affects the career choice and recruitment of younger general physicians.

However, the historical integration of three Japanese primary care societies, the Japanese Medical Society of Primary Care, the Japanese Academy of Family Medicine, and the Japanese Society of General Medicine, into a new organization (the name remains yet undecided), occurred in August 2009. This organization aims to establish a comprehensive certification system and develop the specialty of primary care in Japan. Although still in its infancy, it is certainly beginning to sow the seeds of fostering the next generations of Japanese primary care physicians.

## Competing interests

The authors declare that they have no competing interests.

## Authors' contributions

MMu designed the study. MMu and HK conducted interviews and analysed preliminary data. MMa, the senior researcher, advised on the qualitative methodology and modified the initial results. All three authors discussed, revised, and approved the final result. MMu wrote the initial draft of the manuscript and all three authors revised and finally approved the manuscript. MMu is the guarantor.

## Ethical approval

Formal ethical approval was obtained from The University Ethics Committee at Hokkaido University.

## Disclaimer

Part of this paper was presented at the WONCA 2009 Asia-Pacific Regional Conference, Hong Kong, June 2009.
